# Hyperconnected molecular glass network architectures with exceptional elastic properties

**DOI:** 10.1038/s41467-017-01305-w

**Published:** 2017-10-18

**Authors:** Joseph A. Burg, Mark S. Oliver, Theo J. Frot, Mark Sherwood, Victor Lee, Geraud Dubois, Reinhold H. Dauskardt

**Affiliations:** 1 Department of Materials Science and Engineering, Stanford University, Stanford, CA 94305 USA; 2 Department of Hybrid Polymeric Materials, IBM Almaden Research Center, San Jose, CA 95120 USA

## Abstract

Hyperconnected network architectures can endow nanomaterials with remarkable mechanical properties that are fundamentally controlled by designing connectivity into the intrinsic molecular structure. For hybrid organic–inorganic nanomaterials, here we show that by using 1,3,5 silyl benzene precursors, the connectivity of a silicon atom within the network extends beyond its chemical coordination number, resulting in a hyperconnected network with exceptional elastic stiffness, higher than that of fully dense silica. The exceptional intrinsic stiffness of these hyperconnected glass networks is demonstrated with molecular dynamics models and these model predictions are calibrated through the synthesis and characterization of an intrinsically porous hybrid glass processed from 1,3,5(triethoxysilyl)benzene. The proposed molecular design strategy applies to any materials system wherein the mechanical properties are controlled by the underlying network connectivity.

## Abstract (web summary)

Organic—inorganic glasses can possess unique properties and functionalities, but their poor mechanical strength and stiffness typically limit their applicability. Here the authors demonstrate that inducing hyperconnectivity into silicon-based glass networks endows them with exceptional elastic stiffness.

## Introduction

Hybrid organic–inorganic glasses are a particularly important class of nanomaterials as the combination of organic and inorganic species at the molecular scale leads to novel properties and functionalities. The strength and stiffness of these intrinsically porous and generally brittle materials scale with density, which is controlled by the underlying molecular structure and network connectivity^1–4^. However, designing hybrid glasses to maintain high levels of mechanical strength and stiffness remains a significant challenge that can ultimately limit the integration of these materials into emerging nanotechnologies ranging from microelectronics to antireflective coatings and molecular sieves^5–8^.

To address this challenge, we have exploited the fundamental dependence of the elastic properties on network connectivity by developing a hyperconnected network architecture, wherein the connectivity of a silicon atom within the network extends beyond its chemical coordination number, resulting in exceptional mechanical properties. We initially predicted the elastic properties of a model 1,3,5-benzene hyperconnected glass through molecular dynamic (MD) simulations and subsequently calibrated the model via synthesis and characterization of an intrinsically porous hybrid glass processed from 1,3,5(triethoxysilyl)benzene. Importantly, hyperconnected network architectures represent a new nanoscale design principle to attain nanomaterials with remarkable mechanical properties that are fundamentally controlled by incorporating network connectivity into the intrinsic molecular structure.

## Results

### The superlinear relationship between the modulus and network connectivity

The primary strategy for enhancing the elastic properties of molecular glasses is to increase the network connectivity through processing routes such as post-deposition curing to create more bridging bonds between silicon atoms^9–12^ and the use of bridged precursors in place of those containing terminal organic groups^13–19^. These strategies are based on the fact that in silicate and organosilicate glasses, the elastic modulus (*E*) and bulk modulus (*K*) have a superlinear dependence on the mean silicon network connectivity^1,20^, *m*_Si_:

1 $$E,K \propto \left( {m_{{\mathrm{Si}}} - 2.4} \right)^n$$

where *n* is greater than one. The mean silicon network connectivity, *m*_Si_, is the number of silicon nearest neighbors that a silicon atom is connected to via various linear bridges such as oxygen, methylene, ethylene, or other short organic chains, that is, *m*_Si_ is the average number of topological/bonding constraints for a silicon atom. In rigidity theory^21–27^, mechanical properties are driven by topological constraints and their stiffening/softening behavior with respect to composition^28^. Hybrid glasses are covalently bonded networks that are stressed rigid, wherein the relevant topological constraints are either bridging oxygen or organic bridges derived from the molecular precursor structure. Non-bridging groups (i.e., terminal groups) disconnect the network, do not constrain the silicon atoms, and therefore do not contribute to mean silicon network connectivity. Hence, the nature of the atomic interactions (e.g., bridging vs. terminal) plays a dominant role in the resulting network connectivity^29,30^. Recently, bonding constraints have been shown to control a range of properties including the mechanical, thermal, dopant-dependent, and phase change properties^31–37^.

It is critical to distinguish between chemical coordination number and mean silicon network connectivity, *m*_Si_. Chemical coordination number refers to the number of bonds (covalent, ionic, etc.) in the first coordination sphere of an atom. In our case, these are covalent bonds. Regardless of the molecular model in our work, all silicon atoms have a chemical coordination number of four with four covalent bonds. However, for amorphous hybrid materials, chemical coordination is not sufficient to describe the elastic properties due to the existence of terminal chemical groups that disconnect the network and decrease the stiffness. Thus, *m*_Si_, which only considers bridging constraints, accurately describes the elastic properties of hybrid glasses.

The extent of modulus improvement through increased connectivity for any given precursor structure is limited by the maximum value of *m*_Si_. As silicon is tetravalent with oxygen and carbon, the maximum value of *m*_Si_ for most organosilicate materials is four. In theory, if a precursor was designed that could increase *m*_Si_ to values greater than four, the scaling of modulus with connectivity could be extended, resulting in the formation of a hyperconnected network architecture with exceptional stiffness.

Since *m*_Si_ is the average sum of bridging oxygen and organic bridges, it can be described via two metrics: the condensation degree, *q*, and the intrinsic molecular structure^1^. The condensation degree (the fraction of possible Si–O–Si bonds that have formed), *q*, has a maximum value of unity regardless of precursor structure and generally ranges between 0.70 and 0.85 experimentally. However, increasing the condensation degree >0.85 is experimentally challenging due to steric hindrance phenomena. Interestingly, some hybrids nano-segregate into organic and inorganic regions^38,39^, indicating that there may be compositional and condensation degree limits beyond which organic–inorganic glass formation becomes impossible^40^. Strategies in the materials design community are typically focused on controlling the condensation degree (since linking up a solution of precursor molecules ultimately forms the material), which has lead to design principles focusing on the precursor’s functionality and the reaction conditions. In this work, we increase network connectivity through the intrinsic precursor structure: we propose strategies to increase the number of bridges (topological constraints) in the molecular structure. Thus, we show that the intrinsic precursor structure is a critical parameter toward achieving exceptional mechanical properties.

### Designing a hyperconnected network architecture

**Fig. 1:**
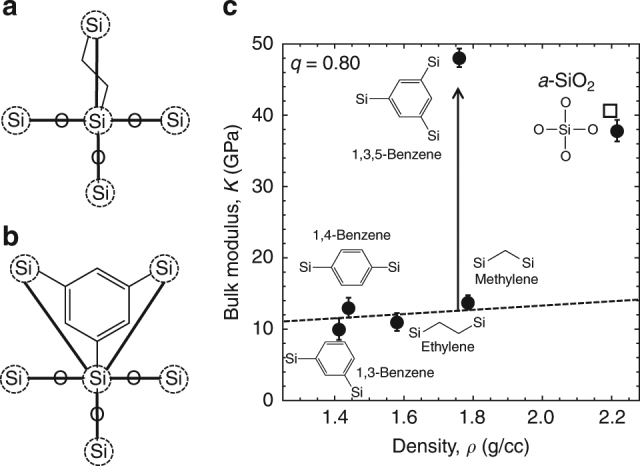
Molecular dynamics simulations predict that hyperconnected glasses have exceptional elastic stiffness. Fully condensed silicon atoms (the condensation degree is *q* = 1.0) have network connectives of *m*_Si_ = 4 and *m*_Si_ = 5 for (**a**) ethylene-bridged glasses and (**b**) 1,3,5-benzene glasses, respectively. **c** The predicted bulk modulus, *K*, as a function of the predicted mass density, *ρ*, for model organosilicate glasses with various organic bridges: ethylene, methylene, 1,4-benzene, 1,3-benzene, and 1,3,5-benzene. The 1,3,5-benzene hyperconnected glasses have a higher stiffness but significantly less density than the experimental value for amorphous SiO_2_ (plotted as the open square)^41–43^. The simulated value for *a*-SiO_2_ shows good agreement with experiment. The error bars denote one standard deviation of the mean of three trials per condition

We present an approach for creating a simple hyperconnected network architecture by using 1,3,5 silyl benzene precursors. The concept of silicon network hyperconnectivity is illustrated in Fig. 1a, b, where the potential silicon network connectivity of a silicon atom in an ethylene-bridged glass and a trifunctional 1,3,5-benzene-bridged glass are shown. We describe the material structure as a network wherein the silicon atoms are nodes connected through a series of atoms and covalent bonds. In glasses with only linear bridges between silicon atoms (e.g., oxygen or ethylene), each silicon atom can connect to a maximum of four nearest silicon neighbors. However, the structure of the 1,3,5-benzene precursor is such that each silicon atom in these networks can potentially connect to five other nearest silicon neighbors. The effect of the benzene ring in the 1,3,5-benzene structure is to connect each silicon atom to two others via carbon bridges that share one common Si–C bond while maintaining the ability of a silicon atom to connect with three others via Si–O–Si bonds. On the basis of Eq. (1), these hyperconnected materials should exhibit superior elastic properties compared to materials with similar chemical composition as *m*_Si_ can have a value as high as five.

Since the silicon network connectivity, *m*_Si_, depends on the condensation degree, *q*, and intrinsic molecular structure, we can explicitly represent *m*_Si_ as a function of both metrics. For the linearly bridged glass models (Fig. 1a), *m*_Si_ is given by

2 $$m_{{\mathrm{Si}}} = 1 + 3q$$

while for the 1,3,5-benzene glass model (Fig. 1b), *m*_Si_ is given by

3 $$m_{{\mathrm{Si}}} = 2 + 3q$$

Thus, for a given condensation degree, the network connectivity can be significantly increased by designing the intrinsic molecular structure to extend the silicon network connectivity.

### Molecular dynamics prediction of the exceptional properties of hyperconnected architectures

We first investigated the prospect of designing molecular architectures to increase network connectivity and thus the mechanical properties with MD simulations. A simulated annealing procedure was used to generate model glass networks of various organosilane precursors with small organic bridges between silicon atoms: methylene, ethylene, 1,4-benzene, 1,3-benzene, and 1,3,5-benzene. Initially, all models were created to have a network condensation degree, *q*, of *q ~*0.80, a typical experimental value. MD was then used to model the bulk modulus of the various glasses. In the models, bonds and angles between the same elements were given the same stiffness regardless of the molecule structure (Supplementary Tables 1–3). We plotted the simulated bulk moduli against the mass density for the various model glasses (Fig. 1c). All of the linearly bridged glass models have a similar bulk modulus ranging between 9.8 and 13.8 GPa: they have the same intrinsic molecular architecture with one organic bridge. In contrast, the 1,3,5-benzene model exhibits a significantly higher modulus of 48.1 GPa at a density similar to the methylene-bridged material. Surprisingly, the 1,3,5-benzene glass has a higher modulus but a significantly lower density than amorphous SiO_2_^41–43^. This exceptional increase in modulus is understood by considering the effect of the intrinsic molecular structure on silicon network connectivity: *m*_Si_ ~3.4 and *m*_Si_ ~4.4 (Eqs. (2) and (3)) for the linear-bridged glass and the 1,3,5-benzene glass, respectively, at *q* ~0.8. The 1,3,5-benzene model is hyperconnected (*m*_Si_ > 4), resulting in an exceptionally high stiffness for its density.

### Calibrating computational models through synthesis and characterization

**Fig. 2:**
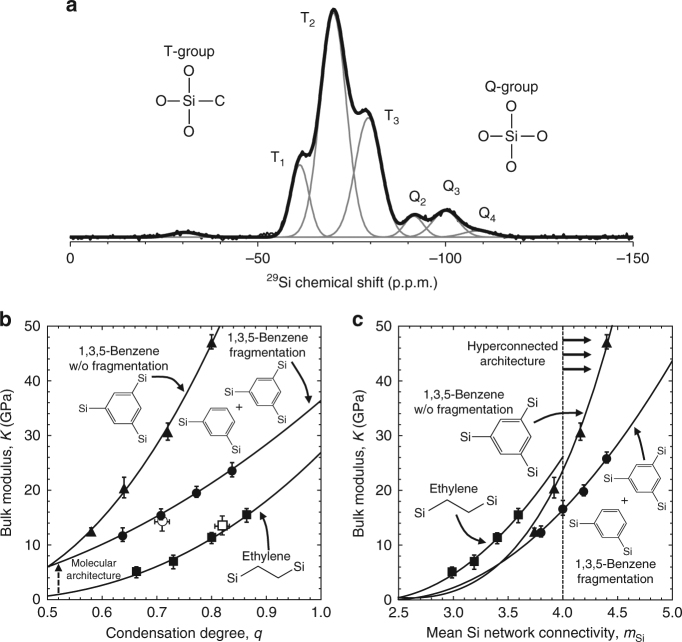
Synthesized glasses calibrate and confirm computational models. **a** The ^29^Si NMR spectra for glass processed from 1,3,5(triethoxysilyl)benzene shows the majority of silicon atoms are coordinated with three oxygen atoms and one carbon atom (T-group configuration), while 10.8% of the silicon atoms are coordinated with four oxygen atoms (Q-group configuration), indicating that some degree of precursor fragmentation occurred, creating SiO_2_ and 1,3-benzene structural units. **b** The bulk modulus, *K*, of the pure 1,3,5-bridged MD model is plotted with respect to condensation degree, *q*. A 1,3,5-benzene glass is synthesized and characterized using NMR and SAWs and is plotted as the open circle. An MD model that includes precursor fragmentation (1,3,5-benzene and 1,3-benzene) is calibrated to the experimental data, where we plot the bulk modulus, *K*, with respect to condensation degree, *q*. The benzene-bridged models with and without precursor fragmentation show a significant increase in bulk modulus, *K*, compared to the ethylene-bridged model (calibrated with experimental synthesis and characterization that is plotted as the open square)^1^ due to the change in the underlying molecular architecture that shifts the modulus to a higher base value (indicated by the dashed arrow). **c** The bulk moduli of the ethylene-bridged, 1,3,5-benzene-bridged, and fragmented 1,3,5-benzene-bridged MD model glasses are plotted against the mean Si network connectivity, *m*_Si_. Values of *m*_Si_ > 4 (the chemical coordination number of Si) correspond to hyperconnected architectures as denoted by the dashed vertical line. The error bars denote one standard deviation of the mean of three trials per condition in **b**, **c**

To calibrate our computational model, a 1,3,5-benzene glass was synthesized via sol-gel chemistry from the precursor 1,3,5(triethoxysilyl)benzene. The film density was 1.52 g/cc, which is significantly lower than fully dense silica (~2.2 g/cc) due to carbon incorporation and intrinsic porosity created during the sol-gel process. The ^29^Si solid-state NMR spectrum for the synthesized glass is shown in Fig. 2a and the network condensation degree was measured to be *q* ~0.72 ± 0.02. As expected, the majority of silicon atoms are in T-group configurations, meaning they are coordinated with three oxygen atoms and one carbon atom. However, 10.8% of the silicon atoms are in Q-group configurations meaning they are coordinated with four oxygen atoms. The presence of Q-groups indicates there is a certain degree of precursor fragmentation during processing where by Si–C bonds are broken, creating SiO_2_ and 1,3-benzene structural units (Supplementary Fig. 1)^44^. We note that the limited fragmentation was due to the precursor structure together with the elevated curing temperature for the film and is not strictly generalizable to organosilicates processed via sol-gel. The result of the precursor dissociation is that the glass processed from 1,3,5(triethoxysilyl)benzene is actually a mixture of 1,3,5-benzene, 1,3-benzene, and SiO_2_ structural units in a molar ratio of ~2:1:1, assuming no precursor loses more than one silicon atom.

We can compare our synthesized glass to those with a dielectric constant of *k* ~3.0 (this corresponds to a density of 1.4–1.5 g/cc). The density and number of residual polar groups will be the dominating factors for the dielectric constant. However, for similar types of materials, we approximate equivalent densities to have similar dielectric constants. Optimized organosilicate glasses deposited via plasma-enhanced chemical vapor deposition (PECVD) have an elastic modulus of *E* ~16 GPa with at most 10 atm% carbon^45^. In our case, the carbon content is as high as 36 atm% and the elastic modulus is *E* ~23 ± 0.5 GPa with a density of 1.52 g/cc. We note that our films have significantly higher carbon content and were not optimized as indicated by the precursor fragmentation and the condensation degree of *q* ~0.72, yet we obtain a significantly higher elastic modulus than state-of-the-art PECVD films due to the underlying molecular architecture.

Using our NMR results on the 1,3,5-benzene precursor fragmentation, we built calibrated MD models with the same characteristics as the synthesized glass. The accuracy of the fragmented MD model predictions is confirmed in Fig. 2b, where the synthesized glasses show good quantitative agreement with our computational models^1^. While the fragmented 1,3,5-benzene model has a reduced modulus compared to the pure 1,3,5-benzene model, both models have significantly higher stiffness than the ethylene-bridged model (a state-of-the-art molecular reinforced hybrid glass^46^) for a given condensation degree: the underlying molecular architecture shifts the modulus to a higher base value (Fig. 2b). Importantly, our synthesized films (and the resulting calibrated model with precursor fragmentation) serve as a baseline for future experimental studies to increase the condensation degree and minimize precursor fragmentation in order to achieve the upper bound of the elastic properties set by our pure 1,3,5-benzene model.

To further illustrate the concept of network hyperconnectivity, the model predictions for the bulk moduli of the ethylene-bridged, 1,3,5-benzene-bridged, and fragmented 1,3,5-benzene glasses are plotted against mean silicon network connectivity, *m*_Si_, using Eqs. (2) and (3) (Fig. 2c). The maximum value of *m*_Si_ for the ethylene-bridged precursor is four. For a 1,3,5-benzene glass, the maximum value of *m*_Si_ is five and can therefore extend the scaling of modulus with network connectivity. It is worth mentioning that in both cases the maximum value for the silicon network connectivity is never achieved experimentally due to steric hindrance phenomena that limit the maximum network condensation degree to 80–90%.

### Precursor rigidity

An important consideration for the application of hyperconnected architectures to other materials is the rigidity of the precursor that connects the silicon atoms. For instance, we show that the 1,4-benzene and methylene glasses have equivalent bulk moduli but different densities (Fig. 1c). Thus, incorporating a rigid aromatic group between silicon atoms in the 1,4-benzene glass leads to a higher stiffness per unit volume compared to the methylene glass. When more than two silicon atoms are connected through the aromatic ring and the network becomes hyperconnected, the impact on the mechanical properties of the molecular network is spectacular. As expected, the density of the molecular network increases from 1.44 to 1.76 g/cc for the 1,4-benzene and 1,3,5-benzene glasses, respectively, but the bulk modulus increases by a factor of 5.

**Fig. 3:**
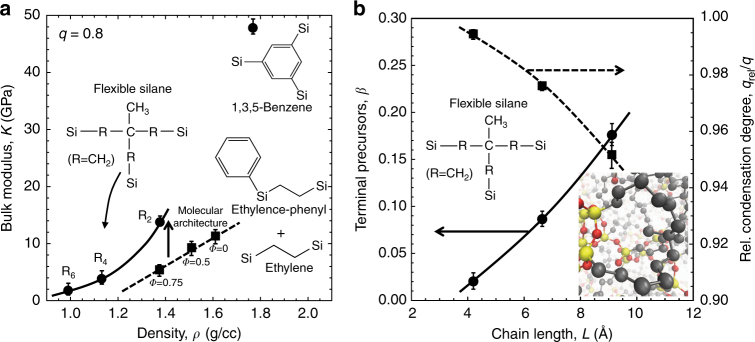
Non-rigid precursors form network terminations and reduce the elastic properties. **a** The predicted bulk modulus, *K*, as a function of the predicted mass density, *ρ*, for model organosilicate glasses: a hyperconnected flexible silane architecture with various side chain lengths (R_2_, R_4_, and R_6_, where R = CH_2_), a 1,3,5-benzene-bridged hyperconnected architecture, and an ethylene-bridge architecture (ethylene) with a fraction of the total precursors, *Φ*, containing a terminal phenyl group (ethylene-phenyl), where *Φ* = 0, 0.5, and 0.75. The circles and squares represent hyperconnected and non-hyperconnected architectures, respectively. The condensation degree, *q*, for all models is *q* = 0.8. The arrow between dashed and solid line shows the difference in the elastic properties between molecular architectures. **b** The fraction of flexible silane precursors, *β*, that form a network termination through a Si–O–Si bond with respect to the side chain length, *L*, of the precursor (R_2_, R_4_, and R_6_, where R = CH_2_), as represented by the circles and solid line. The ratio of the relative condensation degree to the condensation degree, *q*_rel_ */q*, with respect to the side chain length, as represented by the squares and dashed line. The inset is a visualization^67^ of a flexible silane precursor that forms a network termination through a Si–O–Si bond. The error bars denote one standard deviation of the mean of three trials per condition in **a**, **b**

To further study the importance of precursor rigidity, we introduce a hyperconnected flexible silane model and an ethylene-bridged model with phenyl groups (Fig. 3a). We modeled the hyperconnected flexible silane model to have a *m*_Si_ with a maximum value of five: the model contains three silicon atoms per precursor based on a 4-methyl-4-propylheptane structure, where the methyl groups adjacent to CH_2_ groups have been substituted with silicon atoms. We varied the side chain length (R_2,_ R_4,_ and R_6_, where R* = *CH_2_) and plotted the resulting moduli against density with condensation degrees of *q* = 0.8. We see that increasing the chain length decreases the modulus and the density. Importantly, the hyperconnected flexible silane has significantly lower elastic properties than the hyperconnected 1,3,5-benzene. This shows the importance of the fundamental atomic interactions on the resulting mechanical properties. The hyperconnected flexible silane model has flexible angular constraints compared to the rigid angular constraints in the 1,3,5-benzene. This constraint softening contributes to the decrease in the elastic properties.

Beyond the softening of the angular constrains, the flexible silane model formed network terminations by its side chains forming a ring through Si–O–Si bonds (Fig. 3b, inset). To quantify the effect of precursor termination on the condensation degree, we introduce the relative condensation degree, *q*_rel_: the fraction of possible Si–O–Si bonds that form, where each silicon atom must belong to a distinct molecule. Thus, the all Si–O–Si bridging from *q*_rel_ contribute to connecting the molecular network and the resulting elastic properties. Importantly, precursors that form a network termination through Si–O–Si bonds do not contribute to the network connectivity. We show that the fraction of precursors, *β*, that from a network termination increases with respect to increasing side chain length, *L*, in the hyperconnected flexible silane precursors (Fig. 3b). The increase in terminal precursors corresponds to a decrease in *q*_rel_: less Si–O–Si bonds contribute to network connectivity. Thus, we show that the relative condensation degree, normalized by the condensation degree, *q*_rel_/*q*, decreases with increasing side chain length, *L* (Fig. 3b). Precursor rigidity plays an important role in ensuring precursors do not form network terminations and that all Si–O–Si bonds contribute to the network connectivity and the elastic properties. We note that none of the rigid, 1,3,5-benzene precursors formed network terminations.

We also investigate the possibility of steric hindrance effects stiffening the molecular matrix in the case of the rigid, hyperconnected 1,3,5-benzene model. We introduce a varying amount phenyl groups into the ethylene-bridged model at a condensation degree of *q* ~0.8, where the fraction of precursors containing a phenyl group is *Φ* = 0, 0.5, and 0.75. We show that increasing the number of phenyl groups decreases the density and the bulk modulus (Fig. 3a). The elastic properties are fundamentally controlled by the underlying network connectivity. In the case of the ethylene-bridged model with a terminal phenyl group, the network connectivity becomes

4 $$m_{{\mathrm{Si}}} = 1 + 2.5q$$

So introducing a terminal phenyl group decreases the network connectivity (when comparing Eq. (4) to Eq. (2)) and the resulting elastic properties. Thus, rigid terminal phenyl groups do not enhance the elastic properties through steric interactions. Rather, they lead to constraint softening and reduce the network connectivity, resulting in a more compliant matrix compared to non-intrinsically terminated models.

It is important to compare the performance of the hyperconnected flexible silane model to the ethylene-bridged model with terminal phenyl groups. Even at a much lower densities, the hyperconnected flexible silane outperforms the ethylene-bridged model (Fig. 3a). The difference in scaling also shows the importance of the underlying molecular architecture: the hyperconnected flexible silane displays a superlinear dependence of the elastic properties on density, whereas the ethylene-bridged model shows a linear dependence. As the side chain length decreases in the flexible silane, the number of precursors forming network terminations decreases, greatly improving the elastic properties. Introducing rigid precursors, such as the 1,3,5-benzne model, eliminates the possibility of precursors forming network terminations. Thus, the exceptional stiffness of the 1,3,5-benzene glass is the result of both the hyperconnected network and the rigidity of the aromatic group.

### Hyperconnected architectures significantly enhance the modulus of nanoporous glasses

It is well accepted that the incorporation of additional nanoporosity severely degrades the elastic properties and the resistance to processing damage of organosilicates, which can ultimately limit their integration into emerging technologies^47,48^. The sensitivity of these hybrid materials to plasma damage has been addressed in different ways^49,50^, and solutions based on the protection of the nanopores with polymers hold great promise^51–53^. Interestingly, if the polymer used to fill the pores becomes molecularly confined, a unique toughening effect is observed^54^. Nevertheless, we show a promising strategy to significantly improve the mechanical integrity of highly nanoporous organosilicates: designing a hyperconnected precursor to increase the network connectivity.

**Fig. 4:**
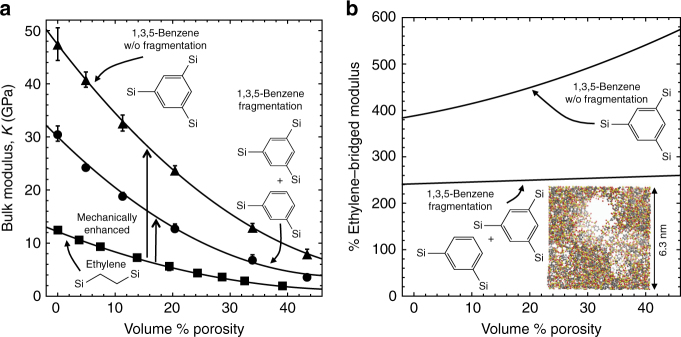
Hyperconnected network architectures enhance the modulus of highly nanoporous glasses. **a** The bulk moduli of pure ethylene-bridged, pure 1,3,5-benzene-bridged, and fragmented 1,3,5-benzene-bridged model glasses are plotted against the volume% porosity. The error bars denote one standard deviation of the mean of three trials per condition. **b** The moduli of the pure 1,3,5-benzene (top) and fragmented 1,3,5-benzene (bottom) glasses are shown as the percentage of the ethylene-bridged modulus as a function of volume% porosity. The inset is a visualization^67^ of a 1,3,5-benzene-bridged nanoporous glass

To introduce additional nanoporosity into our model glasses, we mimic porogen burnout by adding porogen molecules into our simulations and subsequently removing them after our simulated annealing procedure. In Fig. 4a, we show that nanoporosity does degrade the mechanical properties of the pure 1,3,5-benzene, fragmented 1,3,5-benzene, and ethylene-bridged glasses. The modulus tends to 0 as porosity approaches 100%, regardless of the material’s underlying molecular architecture. So the scaling of the much stiffer hyperconnected (1,3,5-benzene) glass models decreases faster than the ethylene-bridged model. However, compared to the nanoporous ethylene-bridged glass, the pure 1,3,5-benzene and fragmented 1,3,5-benzene models continue to enhance the modulus with increasing porosity, reaching ~540 and ~260% of the ethylene-bridged modulus, respectively, at 40% porosity (Fig. 4b). Importantly, our simulations predict that the elimination of precursor fragmentation doubles the bulk modulus of nanoporous materials; however, this proves to be experimentally challenging to realize, and thus synthetic efforts are ongoing.

In conclusion, we have demonstrated that it is possible to extend the connectivity of a silicon atom within the molecular network beyond its chemical coordination number, resulting in a hyperconnected network architecture with exceptional mechanical properties. We first predicted the exceptional elastic stiffness of hyperconnected glasses via MD simulations and subsequently calibrated our results by synthesizing the modeled glass. We show the importance of precursor rigidity by modeling a hyperconnected flexible silane with flexible side chains, which can form network terminations and reduce the stiffness. Hyperconnected architectures also significantly enhance the modulus of highly nanoporous glasses. Importantly, synthesis routes to optimize a material’s mechanical properties (via increasing the network’s condensation degree) are fundamentally limited by the underlying molecular architecture. Thus, hyperconnected architectures represent a new nanoscale design strategy to achieve exceptional mechanical properties for nanomaterials, wherein the elastic properties are fundamentally controlled by the underlying network connectivity.

## Methods

### Interatomic potentials

All molecular modeling was done using LAMMPS^55^. A Stillinger–Weber type potential was developed to both assemble accurate glass networks and provide a quantitatively good approximation of the bulk modulus. The verification of the potential used to assemble the models is described elsewhere^1,56,57^. For the bonded precursors, we used harmonic potentials to model the bonds and angles, and the OPLS and COMPASS potentials to model the dihedral angles (Supplementary Methods)^58,59^. The torsional parameters were determined by fitting to rotational energy profiles obtained from ab initio molecular orbital calculations at the RHF/6–31 G level using Gaussian. The potential parameters are provided in Supplementary Tables 1–5.

### Generating model glasses

To generate the model glasses, various precursor molecules (1,3,5-benzene, 1,3-benzene, 1,4-benzene, methylene-bridged, ethylene-bridged, and Si) and the appropriate number of oxygen atoms were placed randomly into a cubic simulation cell with periodic boundary conditions (Supplementary Fig. 2). All MD simulations were performed with LAMMPS^55^. Initially, a soft potential was used to ensure no atoms were overlapping. Subsequently, a simulated annealing procedure was implemented with NPT dynamics to obtain a low energy structure; the annealing schedule involved cooling the simulation cell linearly from a temperature of 12,000–6000 K over 10,000 one-fs timesteps than quenching from 6000 to 300 K over 100,000 one-fs timesteps. With NPT dynamics, the pressure is controlled and not the volume, so increasing network connectivity increases the density. The pressure profile was chosen such that the densities of the final pressure-free simulation cells representing the experimental glasses were within 5% of the experimentally measured densities^1,56^. The initial pressure was 9 GPa for all simulations and decreased linearly with respect to time until the final pressure-free state was reached. The final simulation cell dimensions varied from 4.8 to 7.2 nm depending on the precursor structure. The accuracy of the local chemical environment of every atom type was verified with the radial distribution functions and coordination numbers described in detail elsewhere^1,57^. The condensation degrees, network connectivities, densities, bulk moduli, and so on were computed on the final equilibrated structures.

### Model condensation degree

The condensation degree of the model networks is controlled by varying the O:Si ratio of the simulation cell. The minimum energy configuration of the simulation, as defined by the potentials, is achieved when all possible Si–O bonds form. Thus, when the O:Si ratio exceeds the stoichiometric ratio for a fully condensed network, non-bridging oxygen atoms are present. The dependence of condensation degree on the O:Si ratio is shown in Supplementary Fig. 3.

### Relative condensation degree

For models with precursors that formed network terminations, we computed the relative condensation degree, where each silicon atom in Si–O–Si bonds must belong to a distinct precursor. We computed the adjacency matrix for Si–C and C–C bonds (since each precursor in our study contained only Si and C atoms). Using the adjacency matrix, we computed the atoms involved in each precursor. If a precursor contained more than one Si–O bond for a given oxygen atom, then the precursor formed a network termination through Si–O–Si bonds and the oxygen atom did not contribute to the condensation degree.

### Bulk modulus

To simulate the bulk modulus, an NPT MD simulation was used to incrementally apply hydrostatic pressure to the simulation cell in a stepwise manner. For each applied pressure, the measured pressure and cell volume were averaged over 20,000 one-fs timesteps. To compute the bulk modulus, *K*, the average pressure, *P*, was plotted against the average volumetric strain (dilatation), Δ, for several applied pressures and the slope was determined with a least-squares fit: *P* = *K*Δ (Supplementary Fig. 4).

### Nanoscale porosity

To introduce nanoporosity into the model network, porogen molecules (which consist of C–C chains with the same interatomic potential and bond angles as the C–C bridge in the ethylene-bridged precursors) were added to the simulation. The porogen molecules had a radius of gyration of 0.4 nm and average diameter of 1.4 nm. The same annealing schedule was used to form a low energy structure. After the simulated annealing step, the porogen molecules were removed from the simulation and the system was equilibrated for 10,000 one-fs timesteps (Supplementary Fig. 5). The condensation degrees, network connectivities, densities, bulk moduli, and so on were computed on the final equilibrated structures.

### Volume% porosity

The volume% porosity was determined by computing the volume fraction of porogen molecules in the simulation cell (each atom type was weighted by its respective van der Waals radius). To validate this approximation, we developed a clustering algorithm to compute the total number of pores in addition to the volume of each pore. The C atoms (associated with the porogen molecules) were assigned to clusters based on a radial cutoff of 5 Å. We computed the convex hull set^60,61^ of each cluster and subsequently the Delaunay triangulation^62^ of each hull set. The constituent tetrahedron volumes in the Delaunay triangulation were summed to compute the volume of each cluster. For low volume% porosities (wherein the clusters were isolated and convex), the convex hull volume matches the volume fraction of porogen molecules (Supplementary Fig. 6). Hence, we use the volume fraction of porogen molecules to approximate the porosity.

### 135TTEB synthesis

1,3,5-Tribromobenzene (98%) purchased from Aldrich was purified by column chromatography over silica gel eluted with hexanes followed by recrystallization from petroleum ether. Triethoxy chlorosilane (95%) was purchased from Gelest. Tetrahydrofuran (THF) was distilled from sodium benzophenone ketyl prior to use. ^1^H-NMR spectroscopy was performed on a Bruker 250 MHz spectrometer. 1,3,5-Tris(triethoxysilyl)benzene was prepared following published procedures:^63,64^ a solution of 1,3,5-tribromobenzene (79.6 mmol) in THF (125 ml) was added over 2 h to a mixture of magnesium turnings (8.10 g, 330.0 mmol) and TEOS (500 ml, 2.24 mol) in THF (500 ml). THF was removed under vacuo and pentane (250 ml) was added. The resulting precipitate was filtered off under nitrogen, and the pentane fraction was isolated. Pentane was removed under vacuo to leave a brown oil. Distillation at 130 °C under 0.04 mmHg gave a clear colorless oil with a 15% yield.

### Thin film synthesis

1,3,5-Tris(triethoxysilyl)benzene of 1 g was dissolved in 4 g of 1-methoxy-2-propanol, and 0.457 g of a 1 M aqueous solution of HNO_3_ was added. The resulting solution was left to age 3 h at room temperature in a closed high-density polyethylene bottle. The solution was spin-coated on 200 mm wafers at 1000 r.p.m. for 45 s and the ensuing films were cured at 400 °C under N_2_ atmosphere for 2 h in a Yield Engineering Systems, Inc. (YES^®^) polyimide bake oven, yielding uniform films ~600 nm thick.

### Sample preparation

1,3,5-Benzene/1,3-benzene/SiO_2_ glass films on 200 mm wafers were scrapped using a metallic razor blade. The resulting powders were dried at 120 °C to eliminate the physisorbed water (if any) and kept under inert atmosphere.

### NMR measurement

Cross-polarization (CP) magic-angle spinning (MAS) ^29^Si NMR spectra were obtained with a Bruker Avance 500 spectrometer operating at 99.353 and 500.113 MHz for ^29^Si and ^1^H, respectively. Ramped-amplitude CP^65^ and two-pulse phase-modulated ^1^H decoupling^66^ were employed. The amplitude of the ^29^Si spin locking field was ramped from 80 to 100% during the contact time and the ^1^H decoupling field was 125 kHz (γ*B*_1_ */*2*π*). The MAS spinning speed was 10 kHz, the contact time was 10 ms, and the relaxation delay was 5 s. Spectra were obtained by averaging 16 k scans on 20–25 mg of sample contained in a 4-mm OD Bruker MAS rotor. The ^29^Si chemical shift was externally referenced using a rotor containing liquid tetramethylsilane.

### Mechanical characterization

The elastic moduli were obtained using surface acoustic wave spectroscopy (SAWS). SAWS studies were performed with a laser-acoustic thin film analyzer (LaWave, Fraunhofer, USA), and acoustic waves were generated by a nitrogen pulse laser with a wavelength of 337 nm and pulse duration of 0.5 ns. The acoustic waves were detected using a transducer employing a piezoelectric polymer film as a sensor. The measured surface wave velocity as a function of frequency was fitted with the theoretical dispersion curve to deduce the elastic modulus. Since the organosilicate glasses are isotropic, we converted the elastic modulus to the bulk modulus, so we could compare values to our simulated bulk modulus: *E* = 3*K*(1 − 2*υ*), where *E* is the elastic modulus, *K* is the bulk modulus, and *υ* is the Poisson’s ratio. For our organosilicate materials, we took *υ* = 0.25.

### Data availability

The authors declare that the data supporting the findings of this study are available within the paper and its supplementary information files.

## Electronic supplementary material

Supplementary Information
