# Supplementary material for: Hyperconnected molecular glass network architectures with exceptional elastic properties
Source: Nat Commun. 2017 Oct 18;8:1019. doi: 10.1038/s41467-017-01305-w (PMC5647325; doi:10.1038/s41467-017-01305-w)
Supplement: Supplementary file 1 — Supplementary Information [file 41467_2017_1305_MOESM1_ESM.pdf]

Supplementary Methods

To model our hybrid organic-inorganic glasses we use two basic inputs: silane precursors and free oxygen atoms. The fundamental structural units for our precursors are shown in Fig. 1c. Hydrogen atoms were modeled implicitly using a united atom approach. Since the precursors have atoms that participate in predefined bonds, angles, and dihedrals, we use bonded potentials to describe these interactions. All other interactions are non-bonded prior to the start of the simulation and are described via the Stillinger-Weber potential. During the simulated annealing process, the free oxygen atoms bond with the silicon atoms on the silane precursors, linking the precursors together and forming the material. Thus, we do not define the network topology prior to structural relaxation. We show this process schematically in Supplementary Fig. 2.

The potentials for the bonds, *U(r)*, and angles, *U(θ)*, within the precursors were modeled as harmonic oscillators:

$$U(r) = k'(r - r_0)^2 \qquad (1)$$

$$U(\theta) = k''(\theta - \theta_0)^2 \qquad (2)$$

where the coefficients k' and r_0 (the equilibrium bond distance) are given in Supplementary Table 1, and the coefficients k'' and θ_0 (the equilibrium bond angle) are given in Supplemental Table 2. The potentials for the dihedral angles, $U(\varphi)$, in the precursors were modeled with a hybrid OPLS and COMPASS dihedral functions[1,2]:

$$U(\varphi)_{OPLS} = k_1(1 + \cos(\varphi)) + k_2(1 - \cos(2\varphi)) + k_3(1 + \cos(3\varphi)) + k_4(1 - \cos(4\varphi)) \qquad (3)$$

$$U(\varphi)_{COMPASS} = k_1(1 + \cos(\varphi - \varphi_1)) + k_2(1 - \cos(2\varphi - \varphi_2)) + k_3(1 + \cos(3\varphi - \varphi_3)) \qquad (4)$$

where the coefficients k_1, k_2, k_3, and k_4 and phase angles φ_1, φ_2, and φ_3 are given in

Supplementary Tables 3,4. We note that we only use the dihedral term in the COMPASS potential, where all other terms are set to zero. To create Si-O bonds during the simulation, an empirical potential of the Stillinger-Weber form[3] was developed as it allowed for easy control of the Si-O bond length with the two-body term, and the O-Si-O, Si-O-Si, and O-Si-C bond angles with the three-body term. The interaction of all non-bonded atom pairs was described by an exponential repulsion function to ensure that atoms did not overlap. The form of the potential energy, $U(r,s,\theta)$, is given by

$$U(r,s,\theta) = \sum_i \sum_{j>i} \phi_2(r_{ij}) + \sum_i \sum_{j\neq i} \sum_{j>k} \phi_3(r_{ij} s_{ik} \theta_{ijk}) \qquad (5)$$

where

$$\phi_2(r) = A\varepsilon \left[B\left(\frac{\sigma}{r}\right)^p - \left(\frac{\sigma}{r}\right)^q \right] \exp\left(\frac{\sigma}{r - a\sigma}\right)$$

and

$$\phi_3(r,s,\theta) = \lambda\varepsilon[\cos\theta - \cos\theta_0]^2 \exp\left(\frac{\gamma\sigma}{r - a\sigma}\right) \exp\left(\frac{\gamma\sigma}{s - a\sigma}\right)$$

For both the two and three body term, σ equals the intercept of potential energy with the r-axis, ε is the characteristic energy, and a sets the cut-off distance with respect to σ (cut-off distance = $a\sigma$). In the two-body term, r is interatomic distance, A controls the energy well depth, and m and n are the exponents that govern the shape of the pairwise energy function. In the three-body term, r and s are interatomic distances, θ is the bond angle, θ_0 is the equilibrium bond angle, λ controls the magnitude of the energy, and γ controls the coupling between the distance and angle energies. The parameterized coefficients are given in Supplementary Table 5.

Supplementary Table 1: Harmonic potential parameters for bonds.

Precursor Types:
Ethylene-bridged Methylene-bridged HFS-R_2 HFS-R_4 HFS-R_6

Atom 1	Atom 2	k' (eV)	r_o (Å)
Si	C	20	1.90
C	C	20	1.54

Precursor Type:
Ethylene-bridged with Phenyl group

Atom 1	Atom 2	k' (eV)	r_o (Å)
Si	C	20	1.90
Si	C_{ar}	20	1.865
C	C	20	1.53
C_{ar}	C_{ar}	20	1.38

Precursor Types:
1,3-Benzene 1,4-Benzene

Atom 1	Atom 2	k' (eV)	r_o (Å)
Si	C_{ar}	20	1.865
C_{ar}	C_{ar}	20	1.42

Precursor Type:
1,3,5-Benzene

Atom 1	Atom 2	k' (eV)	r_o (Å)
Si	C_{ar}	20	1.875
C_{ar}	C_{ar}	20	1.39

Note: C_{ar} refers to aromatic carbon. HFS-R_i refers to the hyperconnected flexible silane model with a side chain length of R_i.

Supplementary Table 2. Harmonic potential parameters for angles.

Precursor Types:
Ethylene-bridged HFS-R_2 HFS-R_4 HFS-R_6

Atom 1	Atom 2	Atom 3	k'' (eV)	θ_o (degrees)
Si	C	C	3.38	109.5

Precursor Types:
Ethylene-bridged with Phenyl group

Atom 1	Atom 2	Atom 3	k'' (eV)	θ_o (degrees)
Si	C	C	3.38	109.5
Si	C_{ar}	C_{ar}	3.38	120
C_{ar}	C_{ar}	C_{ar}	3.38	120

Precursor Types:
1,3-Benzne 1,4-Benzene 1,3,5-Benzene

Atom 1	Atom 2	Atom 3	k'' (eV)	θ_o (degrees)
Si	C_{ar}	C_{ar}	3.38	120
C_{ar}	C_{ar}	C_{ar}	3.38	120

Note: C_{ar} refers to aromatic carbon. HFS-R_i refers to the hyperconnected flexible silane model with a side chain length of R_i.

Supplementary Table 3. OPLS parameters for dihedrals.

Atom 1	Atom 2	Atom 3	Atom 4	k_1 (eV)	k_2 (eV)	k_3 (eV)	k_4 (eV)
Si	C	C	Si	0.375	-0.2	0.625	-0.1
C	C	Si	C_{ar}	0.006063	-0.006386	0.06797	-0.003199
Si	C	C	C	0.08648	-0.03465	0.1369	0.0008816
Si	C_{ar}	C_{ar}	C_{ar}	5.1866	-0.7573	0.1955	-0.04839
*C	C	C	C	0.02604	-0.01523	0.03128	-0.002176

Note: C_{ar} refers to aromatic carbon.
*C-C-C-C has a dihedral angle of 180^0

Supplementary Table 4. COMPASS parameters for dihedrals.

Atom 1	Atom 2	Atom 3	Atom 4	k_1 (eV)	φ_1 (deg)	k_2 (eV)	φ_2 (deg)	k_3 (eV)	φ_3 (deg)
C	Si	C_{ar}	C_{ar}	0	0	0.01072	180	0	0
C_{ar}	C_{ar}	C_{ar}	C_{ar}	5.5263	0	0.05301	-180	-0.4549	0
*C	C	C	C	-0.002123	180	0.006793	-180	0.02489	180

Note: C_{ar} refers to aromatic carbon.
*C-C-C-C has a dihedral angle of 60^0

Supplementary Table 5. Stillinger-Weber parameters control the non-bonded interactions.

Atom 1	Atom 2	Atom 3	ε (eV)	σ (Å)	a	λ	γ	$Cos(\theta_o)$	A	B	p	q
Si	Si	Si	0.5	2.98	1.1	0	0	0	500	2	0	0
Si	O	O	0.25	1.46	2.5	2.22E7	10	-0.333	82.67	1	12	6
Si	O	C	0.25	1.46	2.5	2.22E7	10	-0.333	0	0	0	0
Si	C	O	0.25	1.46	2.5	2.22E7	10	-0.333	0	0	0	0
Si	C	C	0.5	3.9	1.1	0	0	0	500	2	0	0
O	Si	Si	0.25	1.46	2.5	3.64E7	10	-0.819	82.67	1	12	6
O	O	O	0.5	2.66	1.1	0	0	0	500	2	0	0
O	C	C	0.5	2.74	1.1	0	0	0	500	2	0	0
C	Si	Si	0.5	3.9	1.1	0	0	0	500	2	0	0
C	O	O	0.5	2.74	1.1	0	0	0	500	2	0	0
C	C	C	0.004	4.2	1.2	0	0	0	8	1	12	0

Note: Atom 1 is the center atom in the 3-body interaction. All other interactions are non-interacting – parameters are zero.

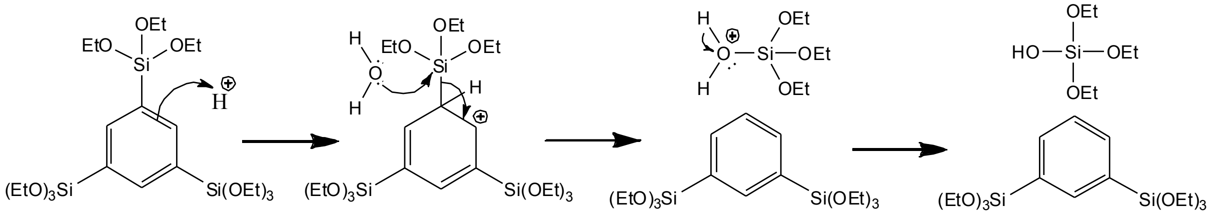

Supplementary Figure 1: Cleaving of the Si-C in 135TTEB to form the 1,3-benzene structural unit and the Q-group silicon. The intermediate carbocation is stabilized by the β-silicon effect.

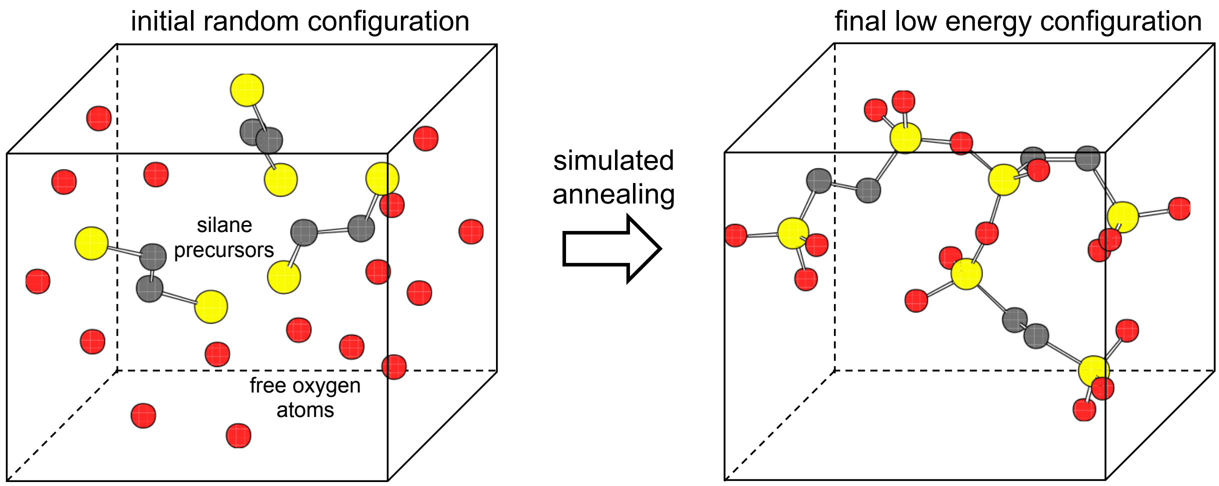

Supplementary Figure 2: Modeling Hybrid Organic-Inorganic Materials. To generate model glasses, silane precursors and free oxygen atoms are randomly placed into a cubic simulation cell with periodic boundary conditions. Bonded potentials describe the bonds, angles and dihedrals in the precursors. The interactions between atoms that are not bonded prior to the simulation are described using a Stillinger-Weber form. After using a soft potential to push apart overlapping atoms, a simulated annealing procedure is implemented to form Si-O bonds and create a low energy configuration.

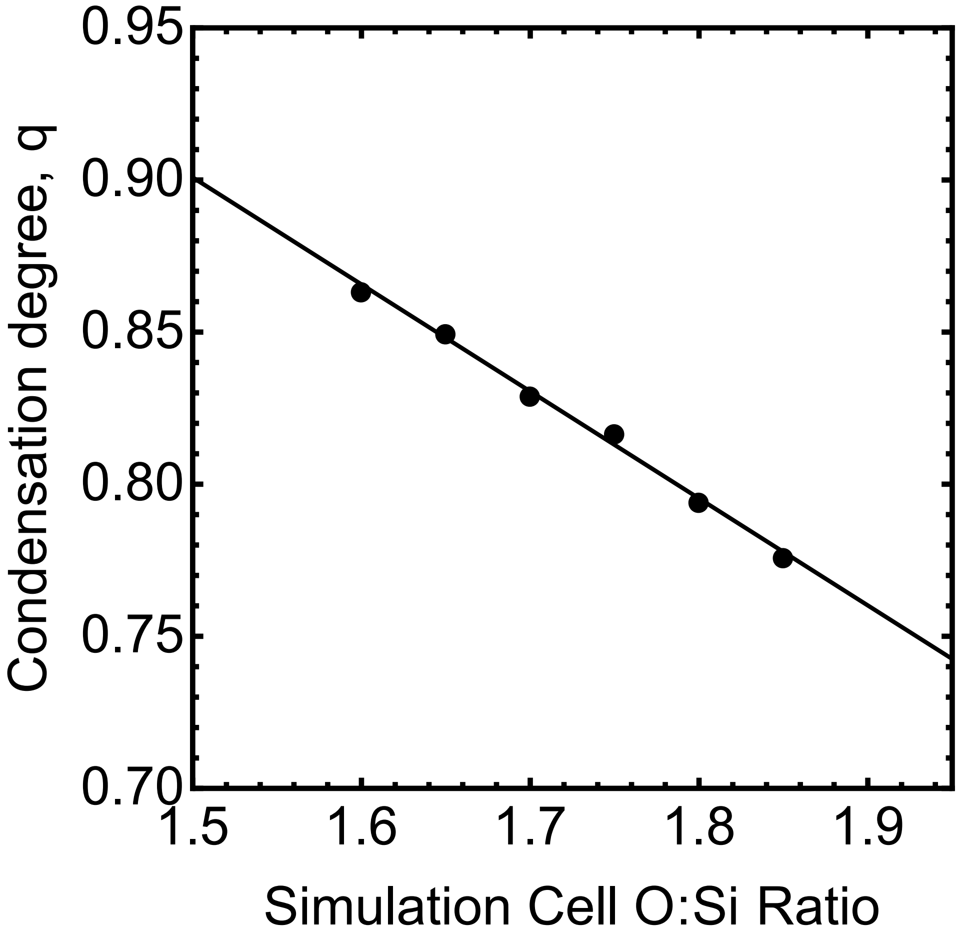

Supplementary Figure 3: The O:Si ratio controls the condensation degree. The number of oxygen atoms introduced relative to silicon atoms into the simulation cell controls the ratio between Si-O (non-bridging oxygen) and Si-O-Si (bridging oxygen) bonds that form. If the O:Si ratio was greater than the stoichiometric ratio for a fully condensed glass, then non-bridging oxygen atoms were present. Continuing to increase the O:Si ratio produces more non-bridging oxygen atoms and thus decreases the condensation degree (the fraction of possible Si-O-Si bonds that formed).

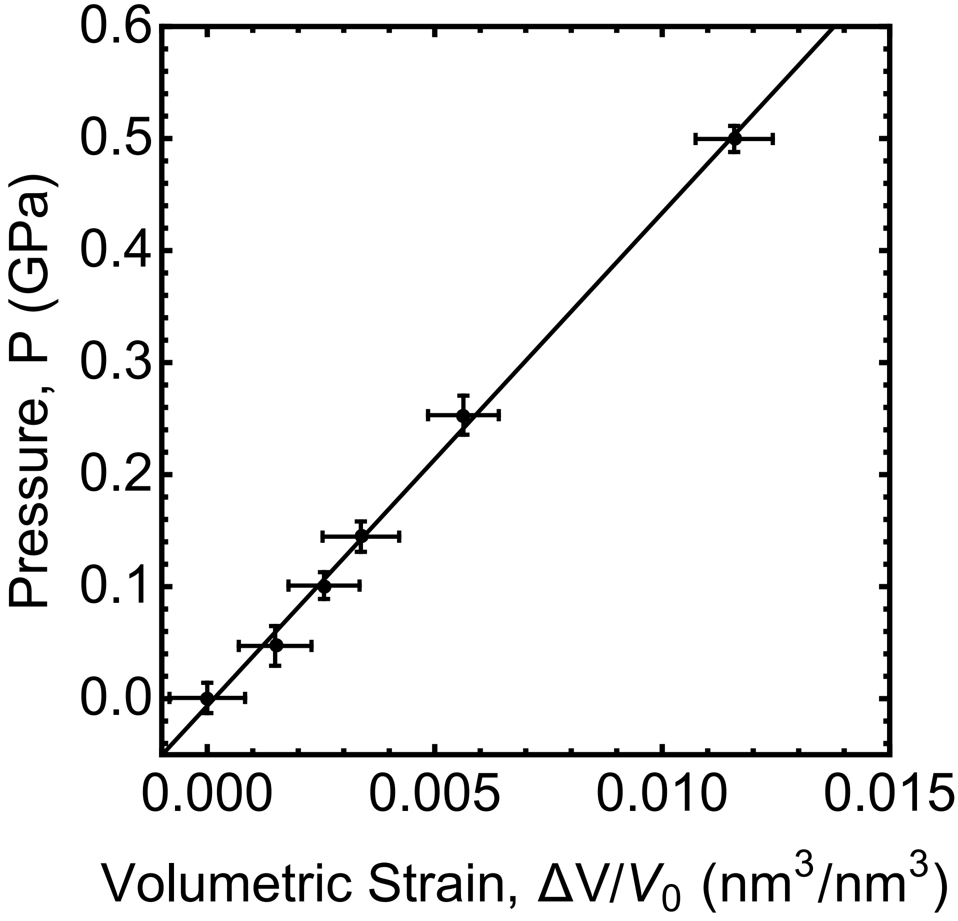

Supplementary Figure 4: Simulating the bulk modulus. To compute the bulk modulus, hydrostatic pressure was incrementally applied to the simulation cell in a stepwise manner so that the pressure and volumetric strain (dilatation) could be averaged over 20,000 timesteps. The slope (bulk modulus) of the average pressure versus the volumetric strain was determined with a least-squares fit. The error bars denote one standard deviation of the mean.

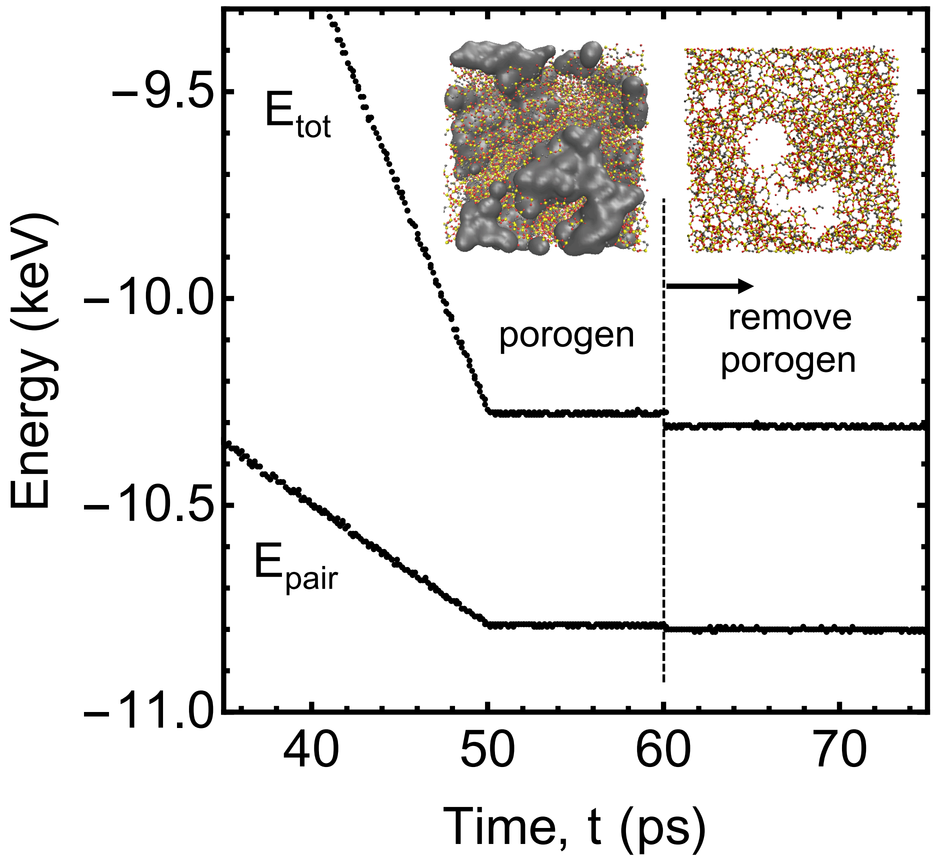

Supplementary Figure 5: Equilibrating the nanoporous matrix after porogen removal. The total energy (potential energy + kinetic energy), E_{tot}, and pairwise energy, E_{pair}, are plotted with respect to time, t. From time t = 0ps to t = 50ps, the simulation cell went through a simulated annealing procedure to reach a low energy configuration. From time t = 50ps to t = 60ps, the simulation cell was equilibrated in the low energy configuration. At time t = 60ps, the porogen molecules were removed from the simulation cell. The dashed vertical line indicates when the porogen molecules were removed. From time t = 60ps to t = 70ps, the system was sufficiently equilibrated after the porogen removal. The inset visualizations show the surface morphology of the porogen molecules (grey) in the hybrid matrix (left) and the resulting nanoporous hybrid matrix after porogen removal (right).

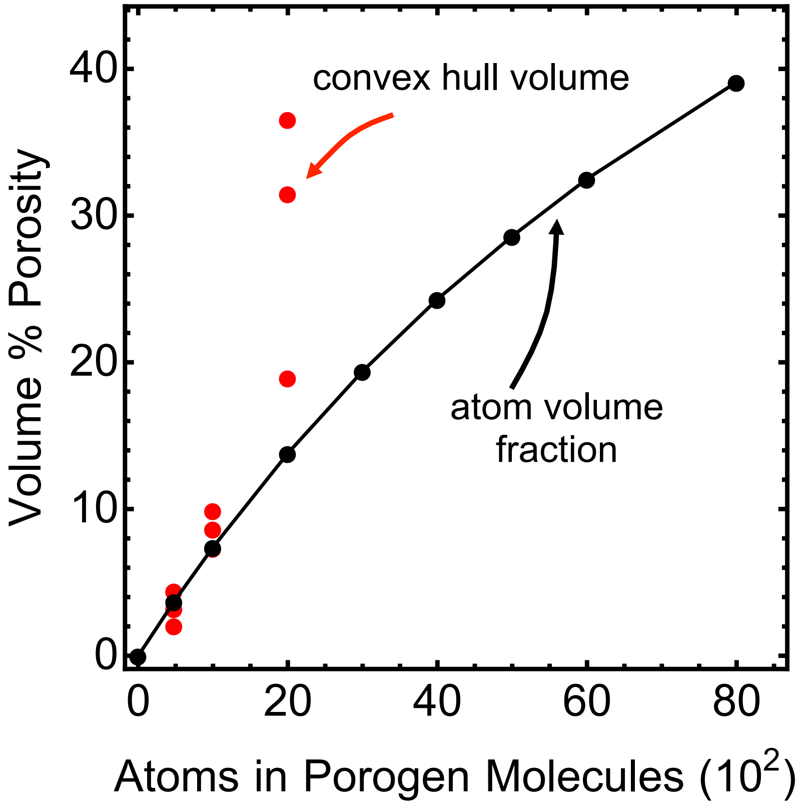

Supplementary Figure 6: Computing the volume % porosity. The volume % porosity is approximated by the fraction of porogen atoms in the simulation (black). The convex hull volumes of the porogen molecule clusters (red) predict the same volume % porosity as the fraction of porogen atoms for nanoscale porosities < 15%. At porosities > 15%, the morphology of the porogen molecule clusters is no longer convex, so the convex hull volumes over estimate the actual pore volumes. Nevertheless, the convex hull volume confirms that the porogen atom volume approximation for the volume % porosity is valid.

Supplementary References:

1. Jorgensen, W. L., Maxwell, D. S. & Rives, J. T. Development and Testing of the OPLS All-Atom Force Field on Conformational Energetics and Properties of Organic Liquids. *J. Am. Chem. Soc.* **118,** 11225–11236 (1996).

2. Sun, H. COMPASS: an ab initio force-field optimized for condensed-phase applications overview with details on alkane and benzene compounds. *J. Phys. Chem. B* **102,** 7338–7364 (1998).

3. Stillinger, F. H. & Weber, T. A. Computer simulation of local order in condensed phases of silicon. *Phys. Rev. B* **31,** 5262 (1985).
